# Professional Excellence

**Published:** 1874-04

**Authors:** W. W. H. Thackston


### AMERICAN JOURNAL

OF

### DENTAL SCIENCE.

Vol. VII. THIRD SERIES--APRIL, 1874. No. 12.

### ARTICLE I.

### *Professional Excellence.*—Concluded.

An Address of Dr. W. W. H. Thackston, President of the Virginia State Dental Association—delivered at its last Annual Meeting.

Comparatively abundant as are now the resources, and perfect the operations in our specialty, the not distant future holds in reserve more astonishing disclosures and more valuable revelations than have yet repaid the diligence and fidelity of the scientist. It is only a question of time, and labor, and *brain*, when we shall not only know the cause and conditions of dental decay, and how to prevent its occurrence, but we shall have a better, a more homogeneous, and manageable material than gold, or tin, or any of the now known amalgams or pastes, with which we may fill, and build out, and restore to permanent usefulness, such teeth as have been or may be permitted to decay and break down. There is a tradition, which I have been unable to trace to any authentic source, but which was asserted as a fact, in an address by the late distinguished Dr. Townsend, of Philadelphia. "That from the ancient burial places of China skulls had been exhumed, containing teeth filled with a white, crystaline substance or material, in a state of perfect preservation, unchanged by service in the mouth, or the thousands of years, and all the influences which had fossilized the bones. And my friend, Dr. F. Y. Clark, late President of the Southern Dental Association, informed me that he had seen fillings inserted by a London dentist who had practiced in the East, of similar character, which had for years withstood the secretions of the mouth, and the office of mastication, and that the only clue to the nature of the material, if indeed it *was* a clue, was the quantity of *egg-shells* which for some unexplained purpose this dentist kept himself supplied with—the suggestion of course being, that the particular form of lime composing the shell was an element or ingredient in the composition of his fillings. More recently I have seen in the Journal the report of a similar filling, examined by Dr. W. George Beers, of Montreal, which had been inserted *fifteen years*, the work of a *native Chinese Dentist*, still perfect, and still preserving and supporting the tooth.

The cement Plombe, the Guillois cement, and all the other preparations of oxychloride of zinc and silex, are but approaches to the discovery of this homogeneous and imperishable material which we need for filling and building out to their original forms the decayed and broken teeth that we now treat with so much labor, and so much uncertainty, with the most precious of all the metals. And so likewise in mechanical or artificial dentistry, "Allen's Continuous Gum Work," while yet imperfect, while exhibiting striking defects and objectionable qualities, is a step, a stride, and a long one, towards all that is desirable in artificial dentures. So of the various anæsthetics, those great boons to suffering humanity, which yet dispense immunity from pain, at the peril of life itself. The time is not distant when we shall have an anæsthetic both certain and effective, and absolutely safe and harmless in its action.

Such are some of the incentives which should stimulate, direct, and energize the efforts of the dentist who aspires to excellence in his profession. As practitioners, as operators, I need not tell you that fidelity to your patients is nothing more, and nothing less, than loyalty to your profession, and devotion to your own interest. Be faithful if need be at the expense of time, and pain, and money, but temper the necessary pain with all the kindness and gentleness which distinguishes a refined and enlightened mind and character. Roughness, indelicacy and rudeness, are qualities least of all befitting the dentist. Abernethy was perhaps not less a great physician, but certainly far less a gentleman and public benefactor, from his boorish manners and indecorous language and deportment. Strive by care and pains-taking, by neatness of person, and cleanliness of instruments, to divest your operations of the dread and disgust with which they are too often regarded. Endeavor to make every operation as perfect as the exercise of all your skill and knowledge will allow, and if possible, make each succeeding operation more perfect than the last.

I am painfully conscious that for the last ten years the circumstances of the South, and of Virginia, especially have been peculiarly unfavorable for the cultivation of excellence in our profession. The war engrossed our thoughts and occupied our time in most instances with unprofessional duties; we were professionally embargoed without and within, and with its close came wide spread and almost universal wreck and ruin. The constrained and enforced neglect of the teeth, yielded a crop of cases affording little opportunity of improvement in practice, and most inadequate compensation for time and labor. A cry and wail went up all over the land for "cheap dentistry," our heart has sickened, and our spirit sunk within us, as we have been daily approached by the cultivated, the refined and appreciative, by the lately wealthy but now beggared—with tears in their eyes, and anguish in their mouths imploring relief; but with the imperative condition that as little as possible must be done, and that little, in the very cheapest manner, and with the cheapest of all available materials. This condition of our

State and southern country has been a great drawback and impediment in the path to professional excellence; but let us continue to work and wait as we have worked and waited in the past; let us continue to work faithfully and under all circumstances do the very best we can; let our motto be "*Patientia et perseverentia vincient Omnia*," and sooner or later our recompense and reward will be assured.

And now, gentlemen, in concluding this address, let me impress upon you not only as a means, but also as an *evidence* of excellence, the cultivation of cordial fraternal relations with each other; let us cherish the "*Esprit de corps*" which binds together and inspires the members of other liberal professions; let all the amenities of life find full and ample illustration in our social and professional intercourse. Scout, and scorn, and repudiate the distrust and jealousy that would seal our lips, lock our libraries, or close our offices and laboratories upon members of our craft and calling. Happily for the cause of science, and the interests of humanity, the day has passed when professional exclusiveness, or personal arrogance can be accepted as evidence of professional excellence. Let us stand shoulder to shoulder, in our efforts for advancement as men of art, and men of science; let us aid and sustain each other, our educational institutions, our authors and our journalists. Let us tread not only all the avenues of strictly professional knowledge, but leave our footprints in the domain of kindred science. Let us strengthen our powers and refine our characters by systematic study—by historic research, by mathematical and logical analysis, and by the cultivation of *belles lettre tastes* and accomplishments. Such exercises are not incompatible, but conducive to the highest excellence in any profession.

Let us build up and permanently establish this state organization, this Virginia Association of Dental Surgeons, and here upon its altar, let us pledge our devotion to whatever duty it may impose, pledge our resolve to perpetuate its existence, to enlarge the sphere of its usefulness, to increase the attractions of its meetings, and to make it an honor to the State, as it now is a credit to science and an honor to our specialty. Let us catch and drink fresh inspiration from these annual assemblies of our best men, and let us bear back to our fields of practice and observation the rich fruitage of this yearly harvest. Let us do all this, and step by step, and year after year, we shall find ourselves approaching that degree of excellence which will reward our highest aspirations, and fully compensate our most earnest and indefatigable labors.

---

